# Hierarchy, Not Lexical Regularity, Modulates Low-Frequency Neural Synchrony During Language Comprehension

**DOI:** 10.1162/nol_a_00077

**Published:** 2022-09-22

**Authors:** Chia-Wen Lo, Tzu-Yun Tung, Alan Hezao Ke, Jonathan R. Brennan

**Affiliations:** Research Group Language Cycles, Max Planck Institute for Human Cognitive and Brain Sciences, Leipzig, Germany; Department of Linguistics, University of Michigan, Ann Arbor, MI, USA; Department of Linguistics, Languages and Cultures, Michigan State University, East Lansing, MI, USA

**Keywords:** neural oscillations, delta rhythms, neural synchronization, language comprehension, syntax, semantics

## Abstract

Neural responses appear to synchronize with sentence structure. However, researchers have debated whether this response in the delta band (0.5–3 Hz) really reflects hierarchical information or simply lexical regularities. Computational simulations in which sentences are represented simply as sequences of high-dimensional numeric vectors that encode lexical information seem to give rise to power spectra similar to those observed for sentence synchronization, suggesting that sentence-level cortical tracking findings may reflect sequential lexical or part-of-speech information, and not necessarily hierarchical syntactic information. Using electroencephalography (EEG) data and the frequency-tagging paradigm, we develop a novel experimental condition to tease apart the predictions of the lexical and the hierarchical accounts of the attested low-frequency synchronization. Under a lexical model, synchronization should be observed even when words are reversed within their phrases (e.g., “sheep white grass eat” instead of “white sheep eat grass”), because the same lexical items are preserved at the same regular intervals. Critically, such stimuli are not syntactically well-formed; thus a hierarchical model does not predict synchronization of phrase- and sentence-level structure in the reversed phrase condition. Computational simulations confirm these diverging predictions. EEG data from *N* = 31 native speakers of Mandarin show robust delta synchronization to syntactically well-formed isochronous speech. Importantly, no such pattern is observed for reversed phrases, consistent with the hierarchical, but not the lexical, accounts.

## INTRODUCTION

Human language is compositional; language users create unbounded and novel phrases and sentences from a finite number of words. This compositional ability is highly structured; words must be combined according to syntactic rules to yield well-formed and interpretable phrases and sentences. Previous studies have narrowed down the neural timing and localization of compositional processing (see Hagoort & Indefrey, 2014; Matchin & Hickok, 2020; Pylkkänen & Brennan, 2019 for reviews). For example, Bemis and Pylkkänen (2011) examined how humans process two-word combinatorial phrases (e.g. “red boat”) vs. non-combinatorial phrases (e.g., “xkq boat”) vs. word lists (e.g., “cup boat”) in magnetoencephalography (MEG) recordings and found that for combinatorial phrases increased activity was elicited at 200–250 ms after the presentation of the second word at the left anterior temporal lobe, unlike for non-combinatorial phrases and word lists. Neufeld et al. (2016) found a greater negativity in the similar time window (184–256 ms) for combinatorial phrases compared to the non-word condition by using the same experimental paradigm in electroencephalography (EEG) recordings. The emerging temporal picture complements functional magnetic resonance imaging (fMRI) studies that narrow down the localization of combinatoric processing. For example, studies have shown greater activation for sentences compared to word lists in brain regions such as inferior frontal gyrus (Pallier et al., 2011; Schell et al., 2017; Zaccarella et al., 2017), posterior superior temporal sulcus (Zaccarella et al., 2017), anterior temporal lobe (Humphries et al., 2006; Matchin et al., 2017), angular gyrus (Humphries et al., 2006; Matchin et al., 2017), and temporal parietal junction (Matchin et al., 2017).

Although many studies have provided neural evidence for *when* and *where* compositional processing takes place, *how* it is actually implemented in neural circuits remains largely underspecified. A growing body of work seeks to develop formal models to account for how computation of hierarchical and compositional processes integrate and modulate neural activity. For example, Martin (2020) argues that linguistic representations may be realized by different patterns of synchronized neural activity while levels of representations are connected by the modulation of neural gain functions. Specifically, a speech envelope segment is recognized as a syllable or phoneme via gain modulation between neural populations that serves to inhibit the process of edge detection of the speech envelope and pass information forward to next stages of lexical and morphosyntactic operations. Repeating this same template at multiple concurrent processes yields a model for a neural architecture that is tuned to linguistic composition at multiple timescales, from phonemes up to sentences. Research in this domain requires examining rhythmic or synchronized neural activity across these different timescales.

Synchronized neural activity, as in the theory developed by Martin (2020), offers one possible response to the “mapping problem” articulated by Poeppel and Embick (2005) and Poeppel (2012). Crucially, the core components of linguistic theories, such as the syntactic operation of Merge, aim to capture representational generalizations, not algorithmic processes; they cannot be directly mapped to neuronal activation. But, it may be feasible to decompose linguistic operations and map them to cross-frequency patterns, which denote the association across multiple frequency bands of neural oscillations (cf. Benítez-Burraco & Murphy, 2019). This leading idea builds on a growing trend that takes synchronized patterns of neuronal circuits as a computational primitive (e.g., Buzsáki & Draguhn, 2004). Consequently, examining patterns of neural synchrony offers a promising avenue to test how neural circuits might work to implement concurrent linguistic processes as continuous speech unfolds.

Consistent with such a model, rhythmic activity at different frequency bands has been linked to distinct stages of language comprehension and speech processing (Arnal et al., 2016; Meyer, 2018). Neural activity in the low gamma band (30–50 Hz) appears to be involved in connecting acoustic fine-structure to discrete phonemic information (Di Liberto et al., 2015; Giraud & Poeppel, 2012). Slower synchronized activity spanning the delta and theta bands (1–4 and 4–8 Hz, respectively) has been linked with the analysis of higher-level syllabic information (Ghitza, 2011; Ghitza & Greenberg, 2009). Rhythmic activity in lower bands has more recently been associated with the processing of more abstract high-level linguistic information. Multiple studies conducting time-frequency analysis have shown evidence that neural activity in the delta band in particular is associated with the processing of syntactic structure (e.g., Bonhage et al., 2017; Kaufeld et al., 2020; Meyer et al., 2016; Meyer & Gumbert, 2018). To give one example, Kaufeld et al. (2020) evaluated the mutual information between neural activity in the delta band and the higher level syntactic content of sentence stimuli, compared to stimuli composed of meaningless words or word lists. They found increased mutual information between EEG signals in the delta band that is specific for sentential stimuli that contain meaningful syntactic structure.

Complementary evidence comes from studies using isochronous speech. Ding et al. (2016) used a frequency-tagging paradigm with sentence stimuli composed from four one-syllable words in Mandarin Chinese. Each monosyllabic word spanned 250 ms, so each sentence was exactly 1 s long. With this design, syllables and words were presented at 4 Hz, two-word phrases at 2 Hz, and sentences repeated at 1 Hz. Crucially, the stimuli were constructed by concatenating individual syllables together, removing prosodic contours at the suprasegmental level (but cf. Glushko et al., 2020). When native speakers of Mandarin listened to these stimuli during MEG recording, neuromagnetic spectral peaks at 1, 2, and 4 Hz were observed. Importantly, for English speakers without Mandarin linguistic knowledge, spectral peaks were observed only at the 4 Hz syllable rate not at the phrasal or sentential rates (2 or 1 Hz).

Ding et al. (2017) replicated these findings using EEG and further demonstrated that these peaks were observed in so-called evoked power (phase-synchronous power changes) and also intertrial phase coherence (consistency of phase-angles across trials), but not in induced power (non-phase-aligned changes in power). This result was also replicated cross-linguistically: English stimuli presented in the same paradigm to English-speaking listeners also elicited entrainment patterns at sentence and phrasal rates.

However, syntactic structure may not be the only explanation for the patterns of delta band entrainment described above. The stimuli used by Ding et al. (2016) were designed such that nouns occurred two times per second (2 Hz) while verbs occurred at 1 Hz. Consequently, the observed signals could reflect neural entrainment to lexical or part-of-speech properties of these words, rather than to hierarchical structure-building (Frank & Yang, 2018).

Against this backdrop, two computational models have been proposed to interpret the functional significance of these peaks; these are summarized in Table 1. Martin and Doumas (2017) proposed a structural account in terms of a time-based binding mechanism. Under this mechanism, lexical-level representations are bound into phrases and, ultimately, sentences by modulations of (a)synchrony between firing units at each respective level. This approach captures the compositional relationship between levels of representation without discarding information from lower levels. Take the adjective phrase “dry fur,” for example. This model encodes semantic features for each word at the lowest layer; word information such as [dry _*adj*_] and [fur _*noun*_] is encoded in the second layer. Artificial neurons in each layer fire asynchronously. A third layer encodes phrase information and will be activated after [dry _*adj*_] and [fur _*noun*_] encodings fire.

**Table 1.** Summary of two accounts and predictions for reversed phrases.

Accounts	Major study	Critical simulation results	Predictions for reversed phrases
Structural account	Martin & Doumas: time-based encoding representations	Sentence: 1, 2, 4 Hz	4 Hz
		Phrase: 2, 4 Hz	
		Word list: 4 Hz	
		Jabberwocky: 1, 2, 4 Hz	
Lexical representation	Frank & Yang: Lexical semantics and POS	Grammatical: 1, 2, 4 Hz	1, 2, 4 Hz
		Phrase: 2, 4 Hz	
		Word list: 4 Hz	

*Note*. Martin and Doumas (2017), Frank and Yang (2018).

Simulations from this model reveal that grammatical sequences (e.g., “dry fur rubs skin”) elicited spectral peaks at 1 Hz, 2 Hz, and 4 Hz, consistent with the experimental results from Ding et al. (2016). Such peaks were also observed in a jabberwocky condition, where nonsense words were combined to retain syntactic relationships but minimize semantic content. This follows as the distinct spectral peaks reflect patterns of synchrony and asynchrony between layers in the model that directly encode structural details. As with the neural signals, word sequences lacking syntactic structure only elicited 4 Hz oscillations in the model.

In contrast to the hierarchical oscillations of Martin and Doumas (2017), Frank and Yang (2018) developed a computational account of these low-frequency spectral peaks by appealing just to sequential patterns of lexical information. They argue that the observed neural synchrony may reflect patterns of words and word categories that are repeated across the stimuli. They tested this hypothesis using a series of simulations in which the stimuli from Ding et al. (2016) were recast as sequences of high-dimensional numerical vectors based on word-to-word co-occurrence in a large corpus of text (*word embedding*; Mikolov et al., 2013). Such vectors capture semantic information through the reasoning that words that are judged to have similar meanings will have more similar vectors; they also encode linguistic regularities like grammatical category of each word, such that two nouns tend to have more similar vectors than a noun and a verb. No further syntactic information for combining phrases and sentences is included in their model. The simulation for both English and Chinese grammatical sentences elicited increased power at 1 Hz, 2 Hz, and 4 Hz. The simulations using Chinese VP stimuli showed increased power at 2 Hz and 4 Hz, but not 1 Hz. Randomly shuffled Chinese monosyllabic words showed increased power at 4 Hz only. These simulation results revealed power spectra similar to that reported by Ding et al. (2016). Frank and Yang (2018) suggest that those neural entrainment patterns may follow from the tracking of lexical or grammatical category sequence information (1 verb/s; 2 nouns/s, etc.).

To summarize, whether neural activity found in the delta range reflects hierarchical information or merely lexical properties remains elusive. Computational models based on either hierarchical structural information or lexical-sequence information have been proposed to account for the neural data from Ding et al. (2016) (see Table 1).

Three previous studies have attempted to tease these two theories apart. Burroughs et al. (2021) recorded EEG while native English speakers listened to isochronous speech that included grammatical adjective-noun phrases, ungrammatical adjective-verb phrases, grammatical mixed phrases, and random syllables. A phrase-level peak was found in the grammatical adjective-noun phrases and mixed phrases, but not in the adjective-verb phrases and random syllables. The results are inconsistent with the lexical representation model, which shows a phrasal-level peak in the adjective-verb condition. A similar conclusion is supported by another recent EEG study using the frequency-tagging approach during a word-monitoring task and a sequence chunking task. Lu et al. (2022) report a 1 Hz sentence-level peak that was weaker in the word list than the sentence condition; they interpret this in support of the hierarchical account.

In contrast, another study appears to support the lexical-sequence account. Kalenkovich et al. (2022) recorded MEG data while Russian speakers listened to isochronous speech that came from one of two different syntactic structures: genitive or dative. The difference was cued by just a single affixal phoneme; all other words and affixes remained the same. This small surface difference affects the underlying phrasal organization of these constructions, and under a direct interpretation of the hierarchical account, these phrasal structures should lead to different patterns of synchrony in isochronous speech. Neural peaks related to sentence, two-word, word, and syllable rates were observed in all conditions, but none of these were modulated by syntactic construction. This is taken to be consistent with the simulated results from the lexical-sequence results.

The above recent studies further show that the functional interpretation of delta rhythms is still under debate. The present study uses reversed phases that preserve semantic information and the regular pattern of parts-of-speech at the lexical level, yet remove any grammatical structure. A lexical-sequence model predicts that isochronous presentation of these reversed stimuli will elicit 1 Hz and 2 Hz peaks because they preserve regular part-of-speech sequences. That is, each sequence still has one adjective, two nouns, and one verb. Computational simulations in which sentences are represented simply as sequences of high-dimensional vectors verify this prediction. In contrast, the structural account predicts no 1 Hz or 2 Hz peaks for reversed phrases, as the original phrase structures are lost. To preview, our EEG data are in line with the structural account such that reversed phrases elicit an oscillatory peak at 4 Hz but not at 1 Hz or 2 Hz; this is inconsistent with the simulated results from the lexical models for these stimuli.

## MATERIALS AND METHODS

This experiment tests whether neural synchronization in the delta band reflects lexical sequence or hierarchical information. If such neural oscillations are modulated by lexical information, specifically, a regular sequence of parts-of-speech (e.g., one verb per second, two nouns per second, etc.), we would expect such synchrony to emerge even when the order of the word sequence is reversed, thereby preserving sequence regularity but disrupting phrase structure (Frank & Yang, 2018). If neural synchrony does depend on hierarchical structure, however, then we would not expect it to emerge for the reversed version of grammatical sentences.

### Participants

Thirty-seven native speakers (22 females, 15 males) of Mandarin Chinese between the ages of 19 and 52 (mean = 27.7) participated in the experiment. They were all right-handed and had normal hearing. They self-reported that they did not have any neurological disorders. They gave informed consent and were reimbursed for their time ($15 per hour in U.S. dollars). Data from six participants were excluded from the analysis due to poor data quality. Thus, data from 31 participants (18 female, 13 males) were included in the final analysis.

### Materials

Experimental items were four-syllable Chinese sequences drawn from 50 sets of four experimental conditions, which are illustrated in Table 2. For condition 1, *Four-syllable sentences* (denoted ABCD) were adapted from Ding et al. (2016), with some modifications. The first two syllables constituted a noun phrase (NP) made up of either Adjective + Noun (e.g., *lao* + *niu* ‘old + cow’) or Noun + Noun (e.g., *shu* + *mu* ‘tree + wood’). The last two syllables constituted a verb phrase (VP) (e.g., *chi* + *cao* ‘eat + grass’). Six items from Ding et al. (2016)’s study were replaced or modified for the following two reasons: (1) Items that do not sound natural for native speakers from either Taiwan or mainland China were replaced with novel sentences; (2) Stimuli using bound morphemes such as *heshang* ‘monk’ and *hudie* ‘butterfly’ cannot be broken down further into Adjective + Noun or Noun + Noun; these were replaced with sentences with free morphemes.

**Table 2.** Stimuli design.

**Condition 1: Four-syllable sentence (ABCD)**	**Condition 2: Semantically-mismatched sequence**
綿 羊 吃 草	軍 孩 奔 草
mian yang chi cao	jun hai ben cao
Cotton sheep eat grass	Soldier child run grass
‘Sheep eat grass.’	
**Condition 3: Two-syllable phrase (ABAB)**	**Condition 4: Reversed phrase (BADC)**
老 牛 青 草	羊 棉 草 吃
lau niu qing cao	yang mian cao chi
Old cattle green grass	Sheep cotton grass eat

The second condition was composed of *Semantically-mismatched sequences*. Following Ding et al. (2016), we randomly replaced each of the four words in the four-syllable sentence condition independently with a new word from another sentence while preserving word position. These replacements were reviewed to ensure that they do not sound meaningful or familiar to native speakers of Mandarin. (This is important as there are many syllables in Mandarin that are completely different in meaning but share the same sounds.)

The third condition was composed of *Two-syllable phrases* of the pattern ABAB. Items in this condition were constructed by extracting the first two words of the four-syllable sentences and pairing them together into NP + NP sequences.

The fourth condition was made up of *Reversed phrases* following the pattern BADC. Here, we reversed the order of the first two words and the last two words from each four-syllable sentence. Crucially, this condition allows us to tease apart lexical from hierarchical synchrony. Similar to four-syllable sentences, this condition includes regular lexical sequences (i.e., noun at 2 Hz and verb at 1 Hz); however, reversed ordering leads to ungrammatical sentences in Mandarin.

All stimuli were recorded using artificial speech synthesis developed by iFLYTek (https://www.xfyun.cn/services/online_tts). Each monosyllabic word was recorded separately to avoid inducing a prosodic contour over the syllable sequences. Each word was compressed to 240 ms, preserving pitch, using the Praat vocal toolkit (Corretge, 2020) in Praat (Boersma & Weenink, 2022) and a 10 ms silence gap was added after each word. As each syllable has a duration of 250 ms, each four-syllable item spans 1 second. Items were further grouped into sequences of 10 that were all drawn from the same condition; each set of 10-second sequences comprised one trial.

**Figure 1.**
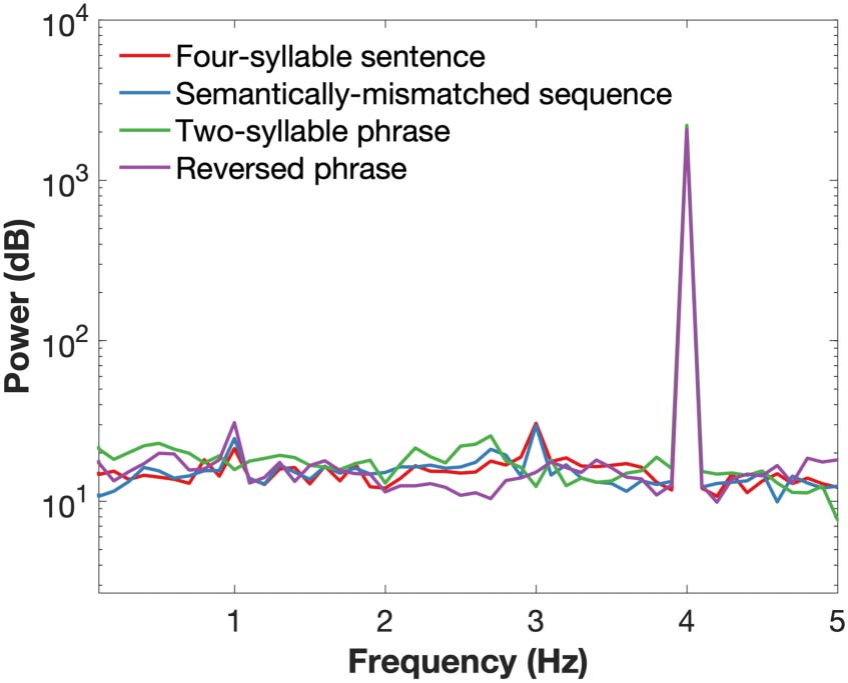
Power spectra for the speech envelope of the stimuli from all four conditions. Only a syllable-level peak at 4 Hz is observed in the speech stimuli.

The power spectrum of the speech stimuli is shown in Figure 1. This was computed using a fast Fourier transform based on the broadband envelope of the stimulus defined by the absolute value of the Hilbert transformation of the stimuli waveforms and then averaged over all 10-second trials for each condition. As expected, only a syllable-level peak at 4 Hz was observed in the acoustic envelope.

Trials were organized into eight blocks, each made up of 20 plausible and 20 implausible trials. Plausible trials were those with grammatical and semantically meaningful phrases, drawn either from Condition 1 (Four-syllable sentences) or Condition 3 (Two-syllable phrases). Implausible trials were drawn from either Condition 2 (Semantically-mismatched sequence) or 4 (Reversed phrases). A given block was made of items from Condition 1 paired with those from Condition 2, or items from Condition 3 paired with those from Condition 4. Trials from each condition were intermixed and presented randomly in each block. Thus, 320 trials were presented to each participant in the whole experiment.

### Procedure

Participants were seated comfortably in front of a computer screen in a quiet room. Prior to the main session, participants were fitted with an electrode cap. Electrodes were also affixed above and below the left eye and electrolyte gel was applied to minimize impedance below 25 kΩ. The setup took approximately 30 minutes. Sound loudness was set for each participant at +45 dB above their hearing threshold (determined using 300 ms 1 kHz tones). Subsequently, 120 1 kHz tones were presented and the auditory-evoked response analyzed to ensure the data quality was sufficient to continue with the experiment.

During the main session, participants were instructed to judge whether a trial included plausible sentences/phrases or not by a button-press. After the button-press, the next trial was played after a delay randomized between 800–1,400 ms (Ding et al., 2016). Stimuli were presented with Psychopy2 (v1.84.2; Peirce, 2007, 2009). Participants were also instructed to avoid frequent blinking and unnecessary body adjustments while the stimuli were presented. Participants had the opportunity to take breaks between each block. Participants had 4 practice trials to become familiar with the procedure of the experiment. The order of blocks was counterbalanced across participants. The main experiment took about 1.5 hr. After the main session, participants washed their hair to remove the electrolyte gel and were debriefed about the goals of the experiment.

### EEG Recording and Data Analysis

EEG data were recorded at 500 Hz from 61 active electrodes (actiCHamp, BrainProducts GMBH) in a 0.01–200 Hz band with online reference to an electrode placed on the left mastoid. Impedances were kept below 25 kΩ. FieldTrip software was used to analyze the data (Oostenveld et al., 2011). Artifacts related to eye blinks were removed via independent component analysis (Jung et al., 2000; Makeig et al., 1995), and remaining trials containing artifacts were removed manually following visual inspection. Following Ding et al. (2017), the first 1-second sentence from each 10-second trial was excluded to avoid potential EEG responses to sound onset.

Data were filtered from 0.1–25 Hz and re-referenced offline to a common average. Synchrony was assessed from 0.5 to 10 Hz at 0.111 Hz intervals; excluding the initial sentence yields 9 seconds of data per trial and thus a frequency resolution of 1/9 = 0.111 Hz. While Ding et al. (2016) assessed synchrony via total power recorded from MEG, the current study follows the analysis from Ding et al. (2017), which separates total power into several components: evoked power, induced power, and intertrial phase coherence.

*Evoked power* reflects the power of EEG responses that is synchronized in both phase and time with speech stimuli. The discrete Fourier transform of the response in trial *n* is denoted as *X*_*n*_(*f*), and *X*_*n*_(*f*) is a complex-value Fourier coefficient. Thus, evoked power is the summation of complex-value Fourier coefficient of trials averaged over the total number of trials *N*.

$$E\left(f\right)=\frac{{\left|\sum_{n}X_{n}\left(f\right)\right|}^{2}}{N}$$ (1)

The 1/f trend in power spectrum was normalized by dividing the value at the target frequency from the average of neighboring values within ±0.5 Hz via Equation 2 adapted from Ding et al. (2017), where *w* represents the neighboring frequency around the target frequency *f*. We adopt this approach to normalization to make our analysis as comparable as possible to that of Ding et al. (2017). (In response to a reviewer query, we also analyzed evoked power using the normalization algorithm proposed by Donoghue et al., 2020, as well as non-normalized evoked power; results are stable regardless of normalization strategy.)

$$En\left(f\right)=\frac{E\left(f\right)}{\sum_{w}E\left(w\right)},\left|w-f\right|<0.5\;\text{Hz},w\neq f$$ (2)

*Intertrial phase coherence* (ITPC) reflects similarities in phase across trials (Cohen, 2014). The summation of cosine and sine values of phase angle *θ*_*n*_ of each complex-value Fourier coefficient is computed and then the square root of the summation is averaged over the total number of trials *N*. (The original formula in Ding et al., 2017, did not take the square root.)

$$R\left(f\right)=\frac{\sqrt{{\left(\sum_{n}\left(\cos\theta_{n}\right)\right)}^{2}+{\left(\sum_{n}\left(\sin\theta_{n}\right)\right)}^{2}}}{N}$$ (3)

*Induced power* reflects the power of EEG responses that is synchronized in time but not phase with the speech stimuli. Induced power is computed from the difference between the complex-value Fourier coefficient per trial and the mean over trials (denoted <*X*(*f*)>) from each trial *n*. Then the summation of difference from each trial is averaged over the total number of trials *N*.

$$I\left(f\right)=\frac{\sum_{n}{\left|X_{n}\left(f\right)-<X\left(f\right)>\right|}^{2}}{N}$$ (4)

For statistical analysis, conditions were compared via a one-way repeated measures analysis of variance (ANOVA) for each measure at each frequency of interest: 1 Hz, 2 Hz, and 4 Hz. A Greenhouse-Geisser correction was applied for calculating *p* values when non-sphericity was indicated by Mauchly’s test.

### Simulations

We conducted a series of simulations to test the predictions of the lexical-sequence account for four-word sentences and reversed phrases under different methodologies for representing word meanings as vectors in a high-dimensional semantic space. Twelve simulated subjects and 50 sentences adapted from Ding et al. (2016) were simulated according to the procedure and code shared by Frank and Yang (2018). First, each word in a sentence was converted to an *N*-dimensional column vector based on the co-occurrence of that word with others in a large corpus of text; this is a word embedding (e.g., Mikolov et al., 2013). These vectors were copied across *M* columns to simulate a word lasting 250 ms, with an onset time *t* drawn from the distribution 𝒰(40, 50) (simulating ear-brain lag). These word representations were concatenated into four-word sentences represented as a *N* × *M* matrix *w*. Gaussian noise with a standard deviation 0.5 was added to each sentence matrix and the discrete Fourier transform was applied to each of *N* rows. Spectral power was then averaged row-wise yielding a single time series for each sentence and each subject, as implemented by Frank and Yang (2018).

This procedure was repeated for both the four-syllable sentences and reversed phrases for each of three different methods for calculating word embeddings: (i) Frank and Yang (2018)’s word vectors for four-syllable sentences (reversed phrases were derived by simply swapping columns; no other parameters were changed), (ii) word embeddings from Wikipedia2vec (Yamada et al., 2020), and (iii) pre-trained Chinese bidirectional encoder representations from transformers (BERT; Cui et al., 2021). Wikipedia2vec was trained from a word-based skip-gram model, an anchor context model, and the link graph model; thus embeddings were learned by predicting the neighboring context from the given words and the link graphs on Wikipedia. Prior literature suggests that Wikipedia2vec trained in this way offers high performance especially on word analogy and text classification tasks (e.g., Yamada et al., 2016; Yamada & Shindo, 2019). In contrast to both the embeddings from Frank and Yang (2018) and Wikipeda2vec (Yamada et al., 2020), BERT is trained with an unsupervised learning and bidirectional approach, which means that the word vectors for the same word may be different depending on the context. Note the Chinese BERT with whole word masking takes the Chinese word segmentation into consideration before training. Thus, the model is trained from masking whole words, instead of word fragments. This model has shown higher performance on various tasks across the sentence and document levels (Cui et al., 2021). We compare word vectors extracted from different models to evaluate the generalizability of Frank and Yang (2018)’s lexical model across alternative methods for representing lexical semantics.

## RESULTS

### Model Simulations

**Figure 2.**
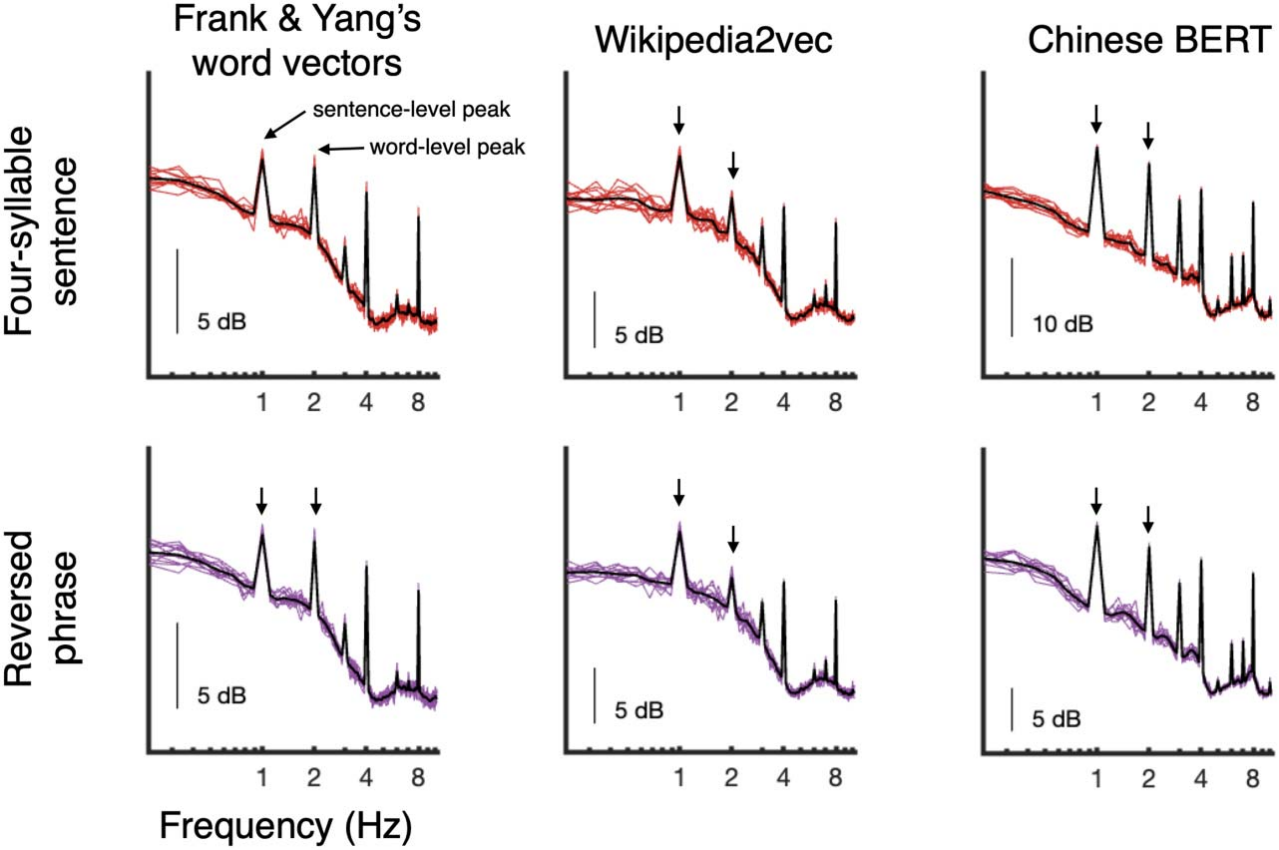
Simulated power spectra for four-word sentences (top) and reversed phrases (bottom) for three different approaches to calculating word embeddings (columns). Colored traces indicate individual simulation trials and black traces indicate the mean spectral pattern. The left-most column shows power spectra simulated using the four-sentence word vectors proposed by Frank and Yang (2018) and their reversed counterpart. Arrows indicate clear spectral peaks at the phrasal (2 Hz) and sentential (1 Hz) level, likely reflecting repeated lexical-level patterns such as part-of-speech information, at these rates. Crucially, these lexical-level patterns are preserved in the reversed phrases. The same pattern is observed when word vectors are calculated using Wikipedia2Vec (middle column) and Chinese BERT (right-most column).

Figure 2 shows the simulated power spectra up to 10 Hz for both four-word sentences and reversed phrases as derived from three separate word embedding representations. As observed by Frank and Yang (2018), four-word sentences showed spectral peaks at 1 Hz and 2 Hz based on the lexical properties of the word sequences alone (top row). Those models carry the prediction that such peaks will also be observed in the novel reversed phrases condition, as the lexical patterns remain unchanged and only hierarchical phrase structure has been disrupted. The experiment tests precisely whether such peaks are also observed in human EEG signals.

### EEG Results

**Figure 3.**
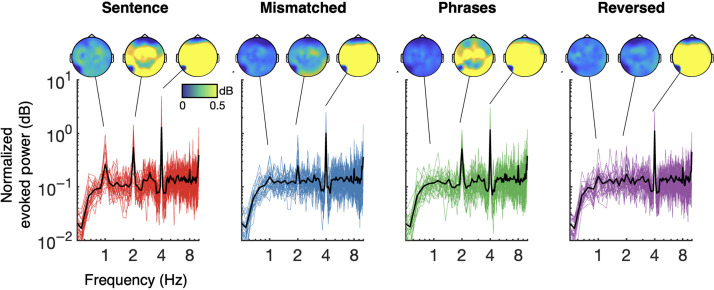
Normalized evoked power (log-scale) for four-word sentences (red), semantically mismatched sentences (blue), two-word phrases (green), and reversed phrases (purple). Colored traces show individual participant data; black traces indicate the group average per condition. Sensor topographies are shown at the 4 Hz syllable/word rate, the 2 Hz phrase rate, and the 1 Hz sentence rate. All conditions show robust entrainment at 4 Hz; phrasal entrainment at 2 Hz is apparent for four-word sentences, two-word phrases, and, to a lesser extent, mismatched sentences. Sentential entrainment at 1 Hz is apparent for four-word sentences only. See main text and Figure 5 for statistical details.

Figure 3 summarizes EEG spectra across all four conditions. Normalized evoked power evidences a 4 Hz “syllable” peak across all conditions. A 2 Hz peak for evoked power was observed for four-syllable sentences and two-syllable phrases, but not for semantically mismatched sentences or, crucially, for reversed phrases. The first three of these results serve to replicate Ding et al. (2016, 2017) by demonstrating that linguistic patterns beyond those explicitly encoded in the acoustic envelope can elicit neural synchrony. The key novel comparison is the result concerning reversed phrases. No 2 Hz “phrase-level” peak was found here, in contrast to predictions from the lexical-sequence model (see simulation results in Figure 2). A similar pattern was also seen for evoked power at 1 Hz: A peak was observed for four-syllable phrases (left-most) but not reversed phrases (right-most). The absence of a 1 Hz peak for semantically-mismatched sentences and two-syllable phrases again replicates findings from Ding et al. (2016). Again, in contrast to predictions of the lexical-sequence model, no 1 Hz peak was observed for reversed-phrases (right-most). Statistical evaluation of these patterns is reported below.

**Figure 4.**
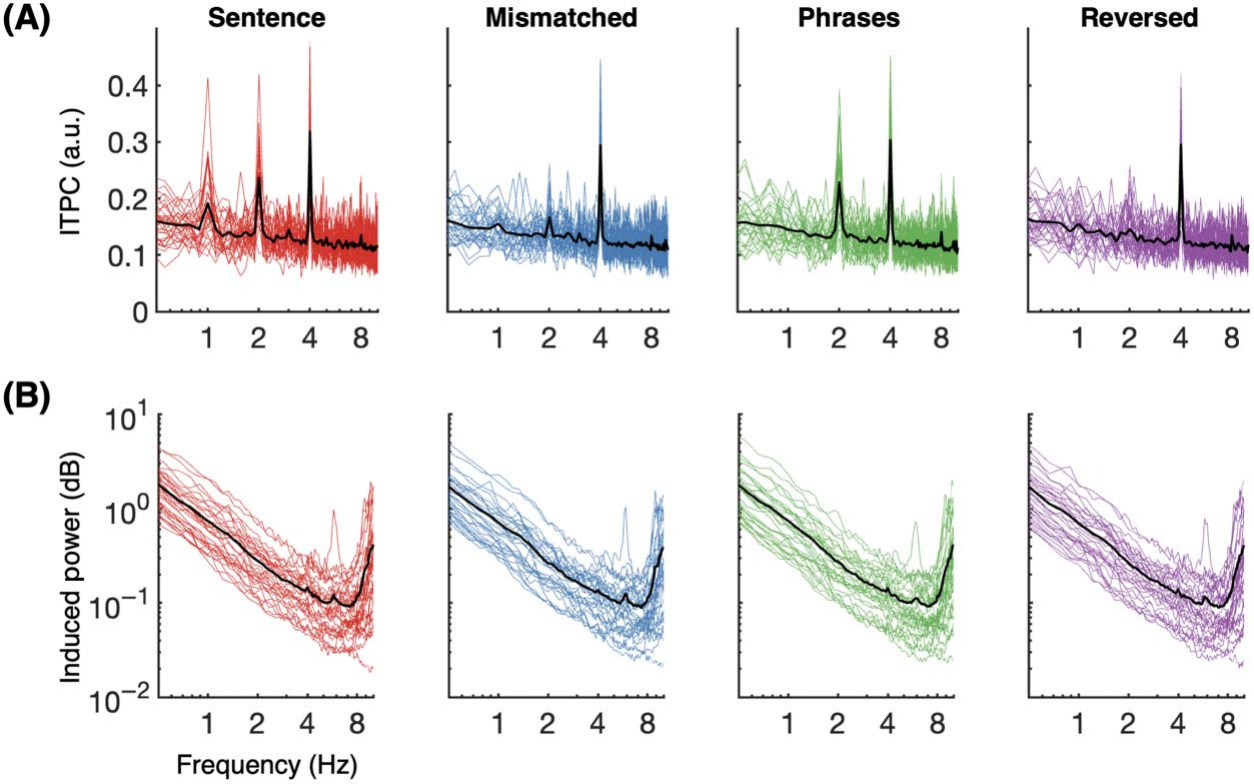
(A) Intertrial phase coherence (ITPC) for four-word sentences (red), semantically mismatched sentences (blue), two-word phrases (green), and reversed phrases (purple). Colored traces show individual participant responses; black traces show the group average per condition. Spectral peaks show phase-alignment at 4 Hz across all conditions, at 2 Hz for four-word sentences and two-word phrases, and at 1 Hz for four-word sentences only. This pattern matches that seen for normalized evoked power. (B) Induced power (log-scale) across four conditions; no relevant spectral patterns are apparent. See main text and Figure 5 for statistical details.

Figure 4 illustrates results for ITPC and induced power, respectively. ITPC results follow the same patterns found for evoked power across all four experimental conditions; this result pattern includes the key absence of 1 Hz and 2 Hz peaks for the reversed phrases condition. No spectral peaks were observed in induced power at any target frequency band (1, 2, or 4 Hz).

**Figure 5.**
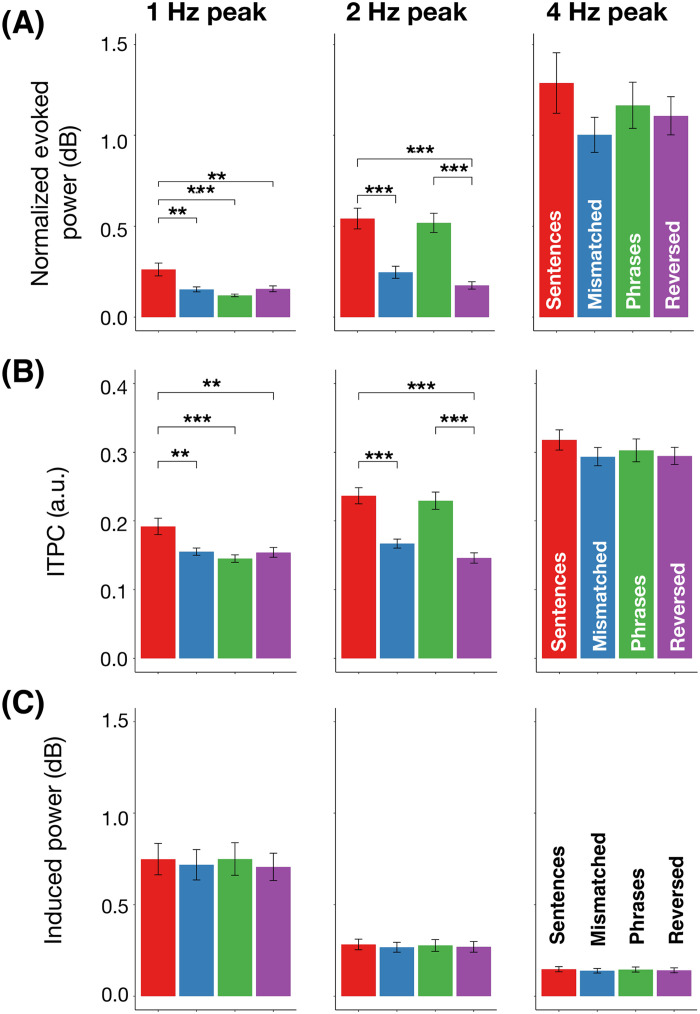
The 1, 2, and 4 Hz spectral activity across four conditions for normalized evoked power (A), Intertrial phase coherence (ITPC) (B), and induced power (C). Error bars indicate ±1 standard error of the mean. Significance code: 0 ‘***’ 0.001 ‘**’ 0.01 ‘*’ 0.05.

Statistical comparisons at each frequency of interest are illustrated in Figure 5. For normalized evoked power, we observed a main effect of condition at 1 Hz (*F*(1.53, 45.9) = 8.16, *p* < 0.01). Post hoc pairwise Tukey’s tests showed a statistically significant difference in the comparison of the four-syllable sentence condition and each of the others (all *p* < 0.01) as well as no significant difference between the semantically mismatched sentences and the phrases (*p* = 0.7), semantically mismatched sentences and the reversed phrases (*p* = 0.99), or between the phrases and reversed phrases (*p* = 0.64). A main effect for condition was also found for the 2 Hz peak (*F*(2.19, 65.7) = 25.97, *p* < 0.001). Post hoc pairwise Tukey’s tests showed statistically significant differences between four-syllable sentences and semantically mismatched sentences (*p* < 0.0001), four-syllable sentences and reversed phrases (*p* < 0.0001), as well as between phrases and reversed phrases (*p* < 0.0001). No statistically significant difference was found in the comparison between four-word sentences and two-word phrases (*p* = 0.97), nor between semantically mismatched and reversed phrases (*p* = 0.51). There was a marginal effect for condition at the 4 Hz syllable peak (*F*(2.22, 66.6) = 2.53, *p* = 0.08).

A nearly identical statistical pattern was observed for ITPC. A main effect at 1 Hz (*F*(1.77, 53.1) = 8.29, *p* < 0.01) was supported by pairwise differences (Tukey’s test) between the four-syllable sentences and all other conditions (all *p* < 0.01); there were no significant differences between semantically-mismatched sentences, phrases, or reversed phrases (all *p* > 0.7). A statistically reliable effect was also found at 2 Hz (*F*(2.16, 64.8) = 30.77, *p* < 0.0001). Post hoc tests revealed significant differences for four-syllable sentences and reversed phrases (*p* < 0.0001), sentences and semantically-mismatched sentences (*p* < 0.0001), phrases and reversed phrases (*p* < 0.0001), as well as between phrases and semantically-mismatched sentences (*p* < 0.0001). No significant difference was found in the comparison between the four-syllable sentences and phrases (*p* = 0.92) nor between the semantically-mismatched and reversed phrases (*p* = 0.27). There was no main effect of condition at 4 Hz (*F*(3, 90) = 1.99, *p* = 0.12).

No statistically reliable effects were observed for induced power (1 Hz: *F*(3, 90) = 1.61, *p* = 0.19; 2 Hz: *F*(1.98, 59.4) = 1.04, *p* = 0.36; 4 Hz: *F*(3, 90) = 2.5, *p* = 0.06).

## DISCUSSION

Low-frequency neural activity in the delta band may become synchronized with abstract linguistic patterns (Ding et al., 2016). We tested between two accounts for the functional interpretation of this synchronization using EEG data and a frequency-tagging experimental protocol where spoken words were presented at a 4 Hz rate with and without syntactic structure. The lexical sequence theory holds that this synchrony emerges due to patterns of sequential lexical or part-of-speech information (Frank & Yang, 2018). The structural account links delta band synchrony with how syntactic structure is encoded across time (Martin & Doumas, 2017); on this account such activity is modulated by hierarchical syntactic information. To tease apart the two accounts, we investigated reversed phrases, which preserve lexical semantics and part-of-speech patterns in comparison to four-word sentences but crucially do not license grammatical structure at the phrasal or sentential level. If delta band neural activity reflects lexical sequence information, reversed phrases should elicit peaks at 1, 2, and 4 Hz, just as seen with regular four-word sentences. Replicating Frank and Yang (2018), we demonstrated with a series of computational simulations that those predictions are robust across a range of embedding strategies for word meaning (see Figure 2). However, if delta band synchrony is modulated by structural information, then reversed phrases (lacking structure) should elicit synchrony only at the 4 Hz rate of monosyllabic words. Inconsistent with the lexical sequence theory and simulations, but consistent with the hierarchical model, EEG data revealed that the reversed phrases elicit peaks at 4 Hz only, in contrast to regular four-word sentences and two-word phrases (see, e.g., Figure 3). These data support the conclusion that neural activity in the delta band reflects the processing of hierarchical information above and beyond lexical-sequence information.

Our data are consistent with the recent report from Burroughs et al. (2021), who tested for neural synchrony by comparing English phrases that followed a grammatical Adj-N phrasal template versus an ungrammatical Adj-V pattern. We replicated their findings that ungrammatical sequences disrupt neural synchrony at the phrasal level using a new manipulation in Mandarin, and also extended their results to the sentential level.

On the other hand, our observations appear to contrast with the conclusions of Kalenkovich et al. (2022), who reasoned that different syntactic structures in Russian should elicit distinct patterns of neural synchrony under hierarchical, but not lexical, accounts. That study and ours used very different strategies for manipulating grammatical structure; crucially our manipulation affects grammatical well-formedness, while the dative and genitive target conditions used by Kalenkovich et al. (2022) are both grammatically acceptable. They reasoned that a hierarchical account would predict greater phrase-level synchrony for genitive structures, where phrases appear at regular intervals, as opposed to dative structures. Yet, similar patterns of neural synchrony were found for the two constructions. The interpretation of this result is highly dependent both on the syntactic analysis of the relevant structures and on the theory of parsing of these structures that underlies online sentence recognition. Both of these facets warrant further study. For example, their particular analysis of datives assumes a ternary-branching structure for verb phrases; a layered verb phrase (Larson, 1988, inter alia) carries distinct predictions about the rate of phrases processed per unit time for these stimuli. The dynamics of the parsing process also bear on how distinct constructions affect synchrony, yet little work has modeled the parsing mechanisms associated with these low-frequency signals (see Brennan & Martin, 2019, for discussion). Progress on sorting out these discrepancies will likely require pairing carefully controlled syntactic manipulations in the mold of Kalenkovich et al. (2022) with explicit models that link parsing with neural mechanisms such as phase resetting (Martin, 2020).

Whether the neural synchrony observed for isochronous speech reflects evoked responses or endogenous oscillatory activities remains under debate (Martorell et al., 2020; Zoefel et al., 2018); our results help to sharpen the issue. In our study, trials built from four-syllable sentences shared the same words as trials built from reversed phrases, and both sequences contained lexical patterns that repeat at 1, 2, and 4 Hz (e.g., 1 verb/second; 2 nouns/second, etc.) If evoked responses are limited to those due to exogenous stimuli, then our results are consistent with the endogenous oscillatory view, perhaps via a phase-reset mechanism (e.g., Martin, 2020). On the other hand, if evoked responses may be attributed to internally generated state transitions, such as recognizing a phrasal node by applying grammatical knowledge, such processing would be time-locked to the isochronous speech rate and thus could give rise to the 1 and 2 Hz patterns of synchrony we observed. That is, the fact that 1 and 2 Hz peaks were only found for regular sentences must be due to endogenous syntactic processing based on the linguistic knowledge of the participant, but whether these signals reflect internally-evoked neural responses or the phase resetting of ongoing oscillatory rhythms remains unknown. Meyer et al. (2019) offers more discussion of how synchronicity might reflect from the combination of external acoustic information and endogenous application of linguistic knowledge.

In addition to the target theoretical question, our results also serve to replicate several earlier observations using frequency tagging and isochronous speech. We replicated with EEG several key results from the MEG study by Ding et al. (2016). As previously reported, four-syllable sentences elicited peaks at 1, 2, and 4 Hz and two-syllable phrases elicited peaks at 2 and 4 Hz, but not 1 Hz. We also found, as with previous reports, that semantically-mismatched sentences elicited absent or attenuated responses at 1 Hz and 2 Hz. While Ding et al. (2016) only investigated neural synchrony using a measure of total power, Ding et al. (2017) separately analyzed evoked and induced power; the former reflects neural activity that is time-locked *and* phase-locked to an external stimulus, while the latter reflects neural activity that is time-locked *but not* phase-locked. They separated out phase locking specifically using ITPC, which measures the phase-consistency neural signals across trials. In line with the EEG findings from English reported by Ding et al. (2017), we observed sentential, phrasal, and syllabic synchrony in evoked power and ITPC, but not induced power. This finding is consistent with patterns of synchrony that reflect a phase-reset mechanism (e.g., Cravo et al., 2011; Kösem et al., 2014).

One concern in the current study is how our results relate to delta band findings from language processing that do not rely on frequency tagging and, more broadly, how results from this less natural experimental protocol might generalize to more naturalistic contexts. Kaufeld et al. (2020) and Coopmans et al. (2022) present one possible avenue forward, where the linguistic properties of more natural stimuli are analyzed in the frequency domain and fit against neural dynamics. Here, rather than isochronous speech, controlled sentences were presented where phrases spanned a narrow temporal window. They observed increased mutual information between EEG signals and the speech envelope within a narrow frequency band defined by the frequency of phrases, but this increase was only observed for structured sentences, not for word lists. Using another strategy, Luo and Ding (2020) tested for oscillatory effects of structure when participants listened to metrical stories, which were made up of pairs of mono- and di-syllabic words in both isochronous speech and natural story listening. They reported no delta band peak in the non-metrical stories, which did not have fixed word onsets and length. These studies provide some insight into the processing of more natural speech, but key questions remain, including how to scale a theory based on relatively narrow-band endogenous rhythms to the higher temporal variation found in quasi-periodic every-day language, and whether the same approach can be applied to longer phrases (and therefore slower neural rhythms).

Other key directions for generalization also remain to be explored. As Martorell et al. (2020) note, it is unclear how neural synchrony of this sort might vary across populations, including in children and patients with aphasia, though see Getz et al. (2018) for an examination of these patterns in a language-learning setting (cf. Maguire & Abel, 2013). Another open question concerns whether these effects generalize across modalities of stimulus presentation (sign vs. speech).

### Conclusion

The current study investigated whether neural activity in the delta band represents the processing of sequence-based lexical items alone or also reflects hierarchical structure. Our findings based on a novel reversed-phrases design are inconsistent with the lexical sequence hypothesis. Only peaks at 4 Hz, but not at 1 Hz and 2 Hz, were elicited in this condition suggesting that low-frequency delta oscillations are not modulated by part-of-speech or word-sequence patterns. This result contrasts with robust tracking of abstract patterns at 1 Hz and 2 Hz for four-word sentences presented at 4 words per second, and for two-word phrases presented at the same rate. That tracking was observed in ITPC and evoked power, but not induced power; this replicates Ding et al. (2016, 2017) and Burroughs et al. (2021) and confirms that cortical tracking of abstract hierarchical information, possibly reflecting a phase-reset mechanism, can be detected robustly across languages with different brain-imaging techniques.

## ACKNOWLEDGMENTS

We thank Samia Elahi for data collection, and audiences from SNL 2019 and AMLaP 2020 for helpful comments.

## AUTHOR CONTRIBUTIONS

**Chia-Wen Lo**: Conceptualization: Equal; Data curation: Lead; Formal analysis: Lead; Investigation: Lead; Methodology: Equal; Visualization: Equal; Writing – original draft: Lead; Writing – review & editing: Equal. **Tzu-Yun Tung**: Conceptualization: Equal; Data curation: Supporting; Methodology: Equal; Writing – review & editing: Equal. **Alan Hezao Ke**: Conceptualization: Equal; Data curation: Supporting; Methodology: Equal; Writing – review & editing: Equal. **Jonathan R. Brennan**: Conceptualization: Equal; Formal analysis: Supporting; Funding acquisition: Lead; Investigation: Supporting; Methodology: Equal; Project administration: Lead; Supervision: Lead; Visualization: Equal; Writing – original draft: Supporting; Writing – review & editing: Equal.

## TECHNICAL TERMS

Neural synchrony: Brain activity that is synchronized to endogenous events.
Frequency tagging: Presenting stimuli rhythmically such that different features occurring at different rates can be used to elicit distinct signatures of neural entrainment or synchrony.
Neural entrainment: Brain activity that is synchronized to the presentation of exogenous events.
Word embedding: A representation of a word as a high-dimensional numerical vector.

## References

[bib1] Arnal, L. H. , Poeppel, D. , & Girard, A.-L. (2016). A neurophysiological perspective on speech processing. In G. Hickok & S. L. Small (Eds.), The neurobiology of language (pp. 463–478). Elsevier. 10.1016/B978-0-12-407794-2.00038-9

[bib2] Bemis, D. K. , & Pylkkänen, L. (2011). Simple composition: A magnetoencephalography investigation into the comprehension of minimal linguistic phrases. Journal of Neuroscience, 31(8), 2801–2814. 10.1523/JNEUROSCI.5003-10.2011, 21414902 PMC6623787

[bib3] Benítez-Burraco, A. , & Murphy, E. (2019). Why brain oscillations are improving our understanding of language. Frontiers in Behavioral Neuroscience, 13, Article 190. 10.3389/fnbeh.2019.00190, 31551725 PMC6736581

[bib4] Boersma, P. , & Weenink, D. (2022). Praat: Doing phonetics by computer (Version 6.2.09) [Computer software]. https://www.praat.org

[bib5] Bonhage, C. E. , Meyer, L. , Gruber, T. , Friederici, A. D. , & Mueller, J. L. (2017). Oscillatory EEG dynamics underlying automatic chunking during sentence processing. NeuroImage, 152, 647–657. 10.1016/j.neuroimage.2017.03.018, 28288909

[bib6] Brennan, J. R. , & Martin, A. E. (2019). Phase synchronization varies systematically with linguistic structure composition. Philosophical Transactions of the Royal Society B, 375(1791), Article 20190305. 10.1098/rstb.2019.0305, 31840584 PMC6939345

[bib7] Burroughs, A. , Kazanina, N. , & Houghton, C. (2021). Grammatical category and the neural processing of phrases. Scientific Reports, 11(1), Article 2446. 10.1038/s41598-021-81901-5, 33510230 PMC7844293

[bib8] Buzsáki, G. , & Draguhn, A. (2004). Neuronal oscillations in cortical networks. Science, 304(5679), 1926–1929. 10.1126/science.1099745, 15218136

[bib9] Cohen, M. X. (2014). Analyzing neural time series data: Theory and practice. MIT Press. 10.7551/mitpress/9609.001.0001

[bib10] Coopmans, C. W. , de Hoop, H. , Hagoort, P. , & Martin, A. E. (2022). Effects of structure and meaning on cortical tracking of linguistic units in naturalistic speech. Neurobiology of Language, 3(3), 386–412. 10.1162/nol_a_00070 PMC10158633 37216060

[bib11] Corretge, R . (2020). Praat vocal toolkit [Computer software]. https://www.praatvocaltoolkit.com

[bib12] Cravo, A. M. , Rohenkohl, G. , Wyart, V. , & Nobre, A. C. (2011). Endogenous modulation of low frequency oscillations by temporal expectations. Journal of Neurophysiology, 106(6), 2964–2972. 10.1152/jn.00157.2011, 21900508 PMC3234094

[bib13] Cui, Y. , Che, W. , Liu, T. , Qin, B. , & Yang, Z. (2021). Pre-training with whole word masking for Chinese BERT. IEEE/ACM Transactions on Audio, Speech, and Language Processing, 29, 3504–3514. 10.1109/TASLP.2021.3124365

[bib14] Di Liberto, G. M. , O’Sullivan, J. A. , & Lalor, E. C. (2015). Low-frequency cortical entrainment to speech reflects phoneme-level processing. Current Biology, 25(19), 2457–2465. 10.1016/j.cub.2015.08.030, 26412129

[bib15] Ding, N. , Melloni, L. , Yang, A. , Wang, Y. , Zhang, W. , & Poeppel, D. (2017). Characterizing neural entrainment to hierarchical linguistic units using electroencephalography (EEG). Frontiers in Human Neuroscience, 11, Article 481. 10.3389/fnhum.2017.00481, 29033809 PMC5624994

[bib16] Ding, N. , Melloni, L. , Zhang, H. , Tian, X. , & Poeppel, D. (2016). Cortical tracking of hierarchical linguistic structures in connected speech. Nature Neuroscience, 19, 158–164. 10.1038/nn.4186, 26642090 PMC4809195

[bib17] Donoghue, T. , Haller, M. , Peterson, E. J. , Varma, P. , Sebastian, P. , Gao, R. , Noto, T. , Lara, A. H. , Wallis, J. D. , Knight, R. T. , Shestyuk, A. , & Voytek, B. (2020). Parameterizing neural power spectra into periodic and aperiodic components. Nature Neuroscience, 23, 1655–1665. 10.1038/s41593-020-00744-x, 33230329 PMC8106550

[bib18] Frank, S. L. , & Yang, J. (2018). Lexical representation explains cortical entrainment during speech comprehension. PLOS ONE, 13(5), Article e0197304. 10.1371/journal.pone.0197304, 29771964 PMC5957381

[bib19] Getz, H. , Ding, N. , Newport, E. L. , & Poeppel, D. (2018). Cortical tracking of constituent structure in language acquisition. Cognition, 181, 135–140. 10.1016/j.cognition.2018.08.019, 30195135 PMC6201233

[bib20] Ghitza, O. (2011). Linking speech perception and neurophysiology: Speech decoding guided by cascaded oscillators locked to the input rhythm. Frontiers in Psychology, 2, Article 130. 10.3389/fpsyg.2011.00130, 21743809 PMC3127251

[bib21] Ghitza, O. , & Greenberg, S. (2009). On the possible role of brain rhythms in speech perception: Intelligibility of time-compressed speech with periodic and aperiodic insertions of silence. Phonetica, 66(1–2), 113–126. 10.1159/000208934, 19390234

[bib22] Giraud, A.-L. , & Poeppel, D. (2012). Cortical oscillations and speech processing: Emerging computational principles and operations. Nature Neuroscience, 15, 511–517. 10.1038/nn.3063, 22426255 PMC4461038

[bib23] Glushko, A. , Poeppel, D. , & Steinhauer, K. (2020). Overt and covert prosody are reflected in neurophysiological responses previously attributed to grammatical processing. BioRxiv. 10.1101/2020.09.17.301994 PMC9427746 36042220

[bib24] Hagoort, P. , & Indefrey, P. (2014). The neurobiology of language beyond single words. Annual Review of Neuroscience, 37, 347–362. 10.1146/annurev-neuro-071013-013847, 24905595

[bib25] Humphries, C. , Binder, J. R. , Medler, D. A. , & Liebenthal, E. (2006). Syntactic and semantic modulation of neural activity during auditory sentence comprehension. Journal of Cognitive Neuroscience, 18(4), 665–679. 10.1162/jocn.2006.18.4.665, 16768368 PMC1635792

[bib26] Jung, T.-P. , Makeig, S. , Humphries, C. , Lee, T.-W. , McKeown, M. J. , Iragui, V. , & Sejnowski, T. J. (2000). Removing electroencephalographic artifacts by blind source separation. Psychophysiology, 37(2), 163–178. 10.1111/1469-8986.3720163, 10731767

[bib27] Kalenkovich, E. , Shestakova, A. , & Kazanina, N. (2022). Frequency tagging of syntactic structure or lexical properties; a registered MEG study. Cortex, 146, 24–38. 10.1016/j.cortex.2021.09.012, 34814042

[bib28] Kaufeld, G. , Bosker, H. R. , ten Oever, S. , Alday, P. M. , Meyer, A. S. , & Martin, A. E. (2020). Linguistic structure and meaning organize neural oscillations into a content-specific hierarchy. Journal of Neuroscience, 40(49), 9467–9475. 10.1523/JNEUROSCI.0302-20.2020, 33097640 PMC7724143

[bib29] Kösem, A. , Gramfort, A. , & van Wassenhove, V. (2014). Encoding of event timing in the phase of neural oscillations. NeuroImage, 92, 274–284. 10.1016/j.neuroimage.2014.02.010, 24531044

[bib30] Larson, R. K. (1988). On the double object construction. Linguistic Inquiry, 19(3), 335–391.

[bib31] Lu, Y. , Jin, P. , Pan, X. , & Ding, N. (2022). Delta-band neural activity primarily tracks sentences instead of semantic properties of words. NeuroImage, 251, Article 118979. 10.1016/j.neuroimage.2022.118979, 35143977

[bib32] Luo, C. , & Ding, N. (2020). Cortical encoding of acoustic and linguistic rhythms in spoken narratives. eLife, 9, Article e60433. 10.7554/eLife.60433, 33345775 PMC7775109

[bib33] Maguire, M. J. , & Abel, A. D. (2013). What changes in neural oscillations can reveal about developmental cognitive neuroscience: Language development as a case in point. Developmental Cognitive Neuroscience, 6, 125–136. 10.1016/j.dcn.2013.08.002, 24060670 PMC3875138

[bib34] Makeig, S. , Bell, A. J. , Jung, T.-P. , & Sejnowski, T. J. (1995). Independent component analysis of electroencephalographic data. In D. S. Touretzky , M. C. Mozer , & M. E. Hasselmo (Eds.), NIPS 1995: Advances in neural information processing systems 8 (pp. 145–151). MIT Press.

[bib35] Martin, A. E. (2020). A compositional neural architecture for language. Journal of Cognitive Neuroscience, 32(8), 1407–1427. 10.1162/jocn_a_01552, 32108553

[bib36] Martin, A. E. , & Doumas, L. A. A (2017). A mechanism for the cortical computation of hierarchical linguistic structure. PLOS Biology, 15(3), Article e2000663. 10.1371/journal.pbio.2000663, 28253256 PMC5333798

[bib37] Martorell, J. , Morucci, P. , Mancini, S. , & Molinaro, N. (2020). Sentence processing: How words generate syntactic structures in the brain. PsyArXiv. 10.31234/osf.io/3utpv

[bib38] Matchin, W. , Hammerly, C. , & Lau, E. (2017). The role of the IFG and pSTS in syntactic prediction: Evidence from a parametric study of hierarchical structure in fMRI. Cortex, 88, 106–123. 10.1016/j.cortex.2016.12.010, 28088041

[bib39] Matchin, W. , & Hickok, G. (2020). The cortical organization of syntax. Cerebral Cortex, 30(3), 1481–1498. 10.1093/cercor/bhz180, 31670779 PMC7132936

[bib40] Meyer, L. (2018). The neural oscillations of speech processing and language comprehension: State of the art and emerging mechanisms. The European Journal of Neuroscience, 48(7), 2609–2621. 10.1111/ejn.13748, 29055058

[bib41] Meyer, L. , & Gumbert, M. (2018). Synchronization of electrophysiological responses with speech benefits syntactic information processing. Journal of Cognitive Neuroscience, 30(8), 1066–1074. 10.1162/jocn_a_01236, 29324074

[bib42] Meyer, L. , Henry, M. J. , Gaston, P. , Schmuck, N. , & Friederici, A. D. (2016). Linguistic bias modulates interpretation of speech via neural delta-band oscillations. Cerebral Cortex, 27(9), 4293–4302. 10.1093/cercor/bhw228, 27566979

[bib43] Meyer, L. , Sun, Y. , & Martin, A. E. (2019). Synchronous, but not entrained: Exogenous and endogenous cortical rhythms of speech and language processing. Language, Cognition and Neuroscience, 35(9), 1089–1099. 10.1080/23273798.2019.1693050

[bib44] Mikolov, T. , Chen, K. , Corrado, G. , & Dean, J. (2013). Efficient estimation of word representations in vector space. ArXiv, 1301.3781v3. 10.48550/arXiv.1301.3781

[bib45] Neufeld, C. , Kramer, S. E. , Lapinskaya, N. , Heffner, C. C. , Malko, A. , & Lau, E. F. (2016). The electrophysiology of basic phrase building. PLOS ONE, 11(10), Article e0158446. 10.1371/journal.pone.0158446, 27711111 PMC5053407

[bib46] Oostenveld, R. , Fries, P. , Maris, E. , & Schoffelen, J.-M. (2011). FieldTrip: Open source software for advanced analysis of MEG, EEG, and invasive electrophysiological data. Computational Intelligence and Neuroscience, 2011, Article 156869. 10.1155/2011/156869, 21253357 PMC3021840

[bib47] Pallier, C. , Devauchelle, A.-D. , & Dehaene, S. (2011). Cortical representation of the constituent structure of sentences. PNAS, 108(6), 2522–2527. 10.1073/pnas.1018711108, 21224415 PMC3038732

[bib48] Peirce, J. W. (2007). PsychoPy—Psychophysics software in Python. Journal of Neuroscience Methods, 162(1–2), 8–13. 10.1016/j.jneumeth.2006.11.017, 17254636 PMC2018741

[bib49] Peirce, J. W. (2009). Generating stimuli for neuroscience using PsychoPy. Frontiers in Neuroinformatics, 2, Article 10. 10.3389/neuro.11.010.2008, 19198666 PMC2636899

[bib50] Poeppel, D. (2012). The maps problem and the mapping problem: Two challenges for a cognitive neuroscience of speech and language. Cognitive Neuropsychology, 29(1–2), 34–55. 10.1080/02643294.2012.710600, 23017085 PMC3498052

[bib51] Poeppel, D. , & Embick, D. (2005). Defining the relation between linguistics and neuroscience. In A. Cutler (Ed.), Twenty-first century psycholinguistics: Four cornerstones (pp. 103–120). Routledge.

[bib52] Pylkkänen, L. , & Brennan, J. R. (2019). Composition: The neurobiology of syntactic and semantic structure building. In D. Poeppel , G. R. Mangun , & M. S. Gazzaniga (Eds.), The cognitive neurosciences (pp. 859–868). MIT Press.

[bib53] Schell, M. , Zaccarella, E. , & Friederici, A. D. (2017). Differential cortical contribution of syntax and semantics: An fMRI study on two-word phrasal processing. Cortex, 96, 105–120. 10.1016/j.cortex.2017.09.002, 29024818

[bib54] Yamada, I. , Asai, A. , Sakuma, J. , Shindo, H. , Takeda, H. , Takefuji, Y. , & Matsumoto, Y. (2020). Wikipedia2Vec: An efficient toolkit for learning and visualizing the embeddings of words and entities from Wikipedia. ArXiv, 1812.06280v3. 10.48550/arXiv.1812.06280

[bib55] Yamada, I. , & Shindo, H. (2019). Neural attentive bag-of-entities model for text classification. In Proceedings of the 23rd conference on computational natural language learning (CoNLL) (pp. 563–573). Association for Computational Linguistics. 10.18653/v1/K19-1052

[bib56] Yamada, I. , Shindo, H. , Takeda, H. , & Takefuji, Y. (2016). Joint learning of the embedding of words and entities for named entity disambiguation. In Proceedings of the 20th SIGNLL conference on computational natural language learning (pp. 250–259). Association for Computational Linguistics. 10.18653/v1/K16-1025

[bib57] Zaccarella, E. , Meyer, L. , Makuuchi, M. , & Friederici, A. D. (2017). Building by syntax: The neural basis of minimal linguistic structures. Cerebral Cortex, 27(1), 411–421. 10.1093/cercor/bhv234, 26464476

[bib58] Zoefel, B. , ten Oever, S. , & Sack, A. T. (2018). The involvement of endogenous neural oscillations in the processing of rhythmic input: More than a regular repetition of evoked neural responses. Frontiers in Neuroscience, 12, Article 95. 10.3389/fnins.2018.00095, 29563860 PMC5845906

